# Responses of *Glossina pallidipes* tsetse fly to geosmin, 2-pentanol and carbon dioxide odour cues

**DOI:** 10.1098/rsos.260488

**Published:** 2026-10-28

**Authors:** Benson Wachira, Robert Opiro, Paul Mireji

**Affiliations:** ^1^ Department of Chemistry, Pwani University, Kilifi, Kenya; ^2^ Biotechnology Research Institute - Kenya Agricultural & Livestock Research Organization, Muguga, Kenya; ^3^ Department of Biology, Gulu University, Gulu, Uganda; ^4^ Center for Virus Research - Kenya Medical Research Institute, Nairobi, Kenya

**Keywords:** geosmin, semiochemicals, carbon dioxide, tsetse attractants, *Glossina pallidipes*, chemical ecology

## Abstract

Tsetse flies exhibit varying host preferences when seeking blood meals. Volatile organic compounds from preferred hosts such as buffalo and cattle have been used to develop the 3-n-propylphenol, 1-octen-3-ol, p-cresol and acetone (POCA) attractant blend, but its attraction is only about 25% of that of natural hosts, necessitating improved attractants. This study evaluated laboratory and field behavioural responses of *Glossina pallidipes* to geosmin, hexanal, 2-pentanol and carbon dioxide (CO_2_), compounds known to elicit strong responses in other insects. Laboratory two-choice olfactometer assays showed dose-dependent attraction to all tested compounds, with significantly more flies choosing treatment arms than control arms (*p* < 0.05). Field responses to geosmin and CO_2_ were validated using a randomized Latin square design, in which traps baited with either odour captured significantly more tsetse flies than unbaited control traps (*p* < 0.05). These findings identify geosmin, 2-pentanol and CO_2_ as promising odour cues for enhancing POCA attractant blends for *G. pallidipes*. Incorporating these compounds into existing odour-bait systems could improve the effectiveness of environmentally friendly and cost-effective tsetse control strategies.

## 1. Introduction

Vector control plays a key role in the management of vector and vector-borne diseases and has contributed to substantial reduction of transmission and near elimination/eradication of the diseases [1]. In African trypanosomiasis, reducing populations of tsetse flies (*Glossina* spp.) is essential for limiting transmission to humans and livestock. Bait technology (use of odour-baited traps and targets) has proved particularly effective because of its relatively low cost, specificity, community acceptability and ability to curb re-invasion from adjacent infested areas while minimizing environmental contamination [2,3]. These technologies exploit olfactory-mediated host-seeking behaviour of tsetse flies, which rely on chemical cues to locate hosts, mates and suitable habitats [4,5]. Harnessing such behaviour-mediating cues provides powerful opportunities to manipulate tsetse populations for surveillance and control.

Extensive research has identified several host-derived attractants, including carbon dioxide (CO_2_), acetone and 1-octen-3-ol, using gas chromatography-linked electrophysiological and behavioural assays [6,7]. Subsequent studies identified phenolic components from fermented cattle and buffalo urine, leading to the development of attractant blends such as 3-n-propylphenol, 1-octen-3-ol, p-cresol and acetone (POCA), widely used for control of savannah tsetse flies [8,9]. However, these synthetic blends typically achieve only partial attraction compared with natural hosts [10,11]. Furthermore, their effectiveness varies across tsetse fly species groups, and to-date, no consistently effective attractants have been identified for riverine species of the palpalis group, which are responsible for much of the transmission of human African trypanosomiasis in West and Central Africa [12,13]. These limitations highlight the need to identify additional behaviourally active odorants to improve and expand semiochemical-based control tools.

Recent studies suggest that compounds not traditionally associated with preferred hosts can also influence tsetse behaviour. For example, structure–activity investigations identified nonanoic acid and 2-nonanone, derived from waterbuck, a non-preferred host, as attractive to savannah tsetse, and demonstrated enhanced attraction when combined with other compounds [14]. These findings suggest that tsetse olfactory systems respond to a broader range of chemical cues than previously recognized, potentially including those originating from microbial and environmental sources.

Microbial and plant-derived volatiles are particularly important in insect chemical ecology. Geosmin (a volatile produced by actinobacteria and fungi) is the major component of the earthy odour of soil and plays diverse ecological roles, attracting insects such as *Aedes aegypti* mosquitoes while repelling others such as *Drosophila melanogaster* [15,16]. Similarly, plant-derived aldehydes such as hexanal and microbial fermentation products such as 2-pentanol are detected by insect olfactory systems and influence host-seeking, feeding and habitat selection behaviours in several *Diptera* species [17,18]. CO_2_ (a respiratory by-product) is one of the most conserved host-associated chemical cues used by hematophagous insects, including tsetse flies, for long-range host detection [19,20]. Despite their ecological relevance in other insects, the behavioural responses of tsetse flies to several of these chemically diverse odorants remain poorly understood.

Recent advances in reverse chemical ecology have shown that ecologically relevant odorants can influence insect behaviour across diverse taxa, providing opportunities to investigate previously unexplored semiochemicals in vector species. Geosmin, an earthy volatile produced by actinobacteria and fungi, mediates habitat selection and oviposition behaviour in mosquitoes while eliciting strong avoidance in *D. melanogater*. Similarly, hexanal is a widespread plant-derived volatile associated with vegetation and host environments, whereas 2-pentanol is a microbial fermentation product characteristic of decomposing organic matter. Although these compounds originate from sources different from cattle-derived attractants, they represent environmentally abundant cues that tsetse flies are likely to encounter during host searching and habitat exploration. We therefore hypothesized that, *G. pallidipes* responds not only to classical host-derived attractants but also to conserved microbial- and plant-derived volatile cues. This reverse chemical ecology framework enables evaluation of ecologically meaningful odorants as potential additions to the existing semiochemical control toolbox. Improved semiochemical-based tools could enhance tsetse control programmes and contribute to reducing the burden of African trypanosomiasis in endemic regions.

## 2. Material and methods

### 2.1. Test odours

Pure (98%–99%) forms of test compounds (delta-nonalactone, 2-pentanol, geosmin and hexanal) were obtained from Sigma-Aldrich (Taufkirchen, Germany). CO_2_ gas was supplied in pressurized cylinders from BOC Gases, Kenya. The POCA, an established tsetse fly attractant blend, was used as a positive control in this study.

### 2.2. Tsetse flies

We obtained 3-day-old teneral male *G. pallidipes* tsetse flies from the Biotechnology Research Institute of the Kenya Agricultural and Livestock Research Organization (BioRI-KALRO), Muguga, Kenya, where we conducted the bioassays. We used the male flies because their behavioural responses have been established to be similar to those of the female flies [21], allowing us to reserve the female flies for colony maintenance. The colony was originally established from wild puparia collected in Uganda (1975) and Zimbabwe (1983). We kept the tsetse flies at 25°C ± 2°C, 75% ± 2% relative humidity (RH) and 12 : 12 hour long-day photoperiod, where we fed them with defibrinated blood from bovines through a silicone membrane (an artificial feeding system) three times a week [22].

### 2.3. Laboratory wind tunnel screening bioassays for responses of *Glossina pallidipes* to hexanal, geosmin or 2-pentanol

We evaluated behavioural responses of *G. pallidipes* in May 2025 in a plexiglass cuboidal wind tunnel (195 × 20 × 20 cm) as described by Wachira *et al.* [23]. We conducted laboratory wind tunnel bioassays under controlled conditions similar to those of the insectary of 25°C ± 2°C and 75% ± 2% RH, performing the experiments during the peak activity periods of *G. pallidipes* (08.00–12.00 and 15.00–17.00 hours) [24]. Briefly, we separately exposed 30 teneral male flies of *G. pallidipes* to 0.025, 0.050 or 0.100 g ml^–1^ of hexanal, geosmin, 2-pentanol or control of delta-octalactone (negative control) in dichloromethane carrier solvent in a two-choice wind tunnel. We conducted all exposures in three independent biological replicates. We delivered test odours and corresponding no-odour controls in carrier solvents into the wind tunnel using pure air (78.09% N₂, 20.95% O_2_ and 0.04% CO_2_) at 12.63 l min^–1^. We observed fly behaviour for 3 minutes, recording departures from the midsection, initial upwind flight direction and final landing/resting positions. To minimize directional bias, we alternated control and odour arms between replicates, and considered flies moving less than 10 cm from the midsection non-responsive and excluded them from analysis. We tested each compound on separate days, monitored residual effects via blank tests and cleaned the tunnel and release cages with water, followed by 70% ethanol after each replicate.

### 2.4. Field responses of tsetse flies to geosmin and CO_2_ odours

We used a randomized Latin square design to assess responses of adult *G. pallidipes* to the treatments in Shimba Hills National Reserve in August, 2025. We selected Shimba Hills National Reserve for the study because *G. pallidipes* is predominant. We deployed NG2G traps [10] at 300 m intervals to minimize spatial interactions. Our field experiment comprised three independent replicates, each consisting of four treatments rotated across four trap sites over four consecutive days, as we described previously [23,25], resulting in 48 trap deployments in total. Our treatments comprised the trap treated with (i) no odour (blank control), (ii) POCA (positive control), (iii) geosmin, or (iv) CO_2_ gas in three independent replicates (conducted in separate independent blocks 1–2 km apart within the study area). We did not advance evaluations of responses of the flies to hexanal or 2-pentanol in the field because our laboratory bioassays identified geosmin as the best-performing novel compound and included CO₂ as a biologically established host cue for field validation. We dispensed geosmin using sealed thin-walled polythene sachets constructed from polyethylene lay-flat tubing (200-µm thick) folded to form a surface area of approximately 50 cm^2^ [25], and separately released acetone from clear glass bottles. We pegged the sachets 25 cm above the ground and 30 cm downwind from the centre of the NG2G traps, and collected trap tsetse fly catches in 24-hour intervals to encompass morning and afternoon bimodal *G. pallidipes* activity peaks [24]. We identified the flies to species level using standard taxonomic keys as described by Pollock [26], and calculated mean release rates of our test material from daily loss in masses, pre- versus post-deployment.

### 2.5. Data analyses

We calculated percentage attraction responses as (*T*–*C*)/(*C*+*T*) × 100, where *T* and *C* represented the number of flies in the treated and control arms of the wind tunnel, respectively. We subjected proportional responses of the tsetse flies to various odours in the laboratory wind-tunnel experiments to analysis of variance (ANOVA) and separated the means by Student–Newman–Keuls (SNK) post hoc analyses. We compared average distances of upwind flight by activated flies in the treated and the control arms using independent unpaired *t*-tests, and log_10_ (*n* + 1) transformed field catches of tsetse flies to normalize the distributions. We separated the effects of site, day and test odour using ANOVA, followed by Fisher's least significant difference post hoc analyses. We conducted statistical analysis using SPSS Version 22 (SPSS, Inc., Chicago, IL, USA) with a confidence level of 95% and de-transformed the mean catches for reporting.

## 3. Results

### 3.1. Laboratory responses of *Glossina pallidipes* to selected test odours

We summarize behavioural responses of *G. pallidipes* to hexanal, geosmin, 2-pentanol, delta-octalactone (negative control) or POCA (positive control) in table 1 . The proportion of *G. pallidipes* that chose the treated arm was significantly (*p* < 0.05) higher than those of the control arm when hexanal, geosmin, 2-pentanol or POCA was dispensed (table 1). The responses of *G. pallidipes* to geosmin or POCA cues were similar (*p* > 0.05; table 1). Responses of *G. pallidipes* to geosmin cues were significantly (*p* < 0.05) higher than similar responses to hexanal or 2-pentanol (table 1). The *G. pallidipes* tsetse flies flew significantly (*p* < 0.05) longer distances within the hexanal, geosmin or 2-pentanol–treated arm, relative to their similar responses in the control arm when dispensed (table 1). This pattern was similar to the responses of the flies to POCA (positive control), with the responses to delta-octalactone (known repellent–negative control) exhibiting the reverse pattern. In all the concentrations we tested, geosmin indicated the greatest difference (*p* < 0.001) in distances flown between the control and treated arms (table 1). *Glossina pallidipes* indicated a dose-dependent behavioural (attraction) response to geosmin, 2-pentanol or hexanal (table 1). We observed increased *G. pallidipes* attraction responses to hexanal, geosmin or 2-pentanol with increasing exposure concentration from 0.025 to either 0.050 or 0.100 g ml^–1^, though the increase was not statistically significant (*p* > 0.05; table 1).

**Table 1.** Responses of *G. pallidipes* to hexanal, geosmin and 2-pentanol. (Raw values are provided in the supplementary material. Note: number of tsetse flies used in each test (*n *= 30 × 3); C, control arm dispensed with CH_2_Cl_2_ carrier solvent; T, treated arm with different odours; ^#^Positive control (POCA); each pair of average distance of upwind flight in C and T were compared by $\chi^{2}$, ^*^p < 0.05, ^**^p < 0.01 and ^**^p < 0.001. Means followed by the same letter (^a^, ^b^ or ^c^) in ‘final resting choice’ are not significantly different (*p* > 0.05, SNK test); ^¥^, dichloromethane solvent.)

dose (g ml^–1^)	test material	average distance of upwind flight (cm ± s.e.)		final resting choice (attraction)
		C	T	(*T*–*C*)/(*C*+*T*)100 ± s.e.
0.00	residual solvent^¥^	59.13 ± 3.31	46.19 ± 2.54	0.00 ± 0.00
	POCA^#^	47.71 ± 4.02	74.34 ± 4.41^*^	54.08 ±0.91^c^
0.025	2-pentanol	26.38 ± 3.15	48.64 ± 3.88^*^	26.67 ± 6.67^b^
	geosmin	21.67 ± 5.92	57.14 ± 3.95^***^	50.04 ± 1.48^c^
	hexanal	28.14 ± 2.94	54.76 ± 5.57^**^	30.37 ± 5.19^b^
	delta-octalactone	55.12 ± 3.63	28.49 ± 2.40^**^	–57.93 ± 5.93^a^
0.050	2-pentanol	23.25 ± 3.94	46.33 ± 6.93^**^	29.19 ± 5.19^b^
	geosmin	21.14 ± 6.19	51.36 ± 5.21^***^	52.85 ± 6.06^c^
	hexanal	25.73 ± 3.52	52.37 ± 2.69^**^	33.44 ± 3.58^b^
	δ-octalactone	57.32 ± 3.47	23.04 ± 2.61^***^	–59.20 ± 3.76^a^
0.100	2-pentanol	21.44 ± 4.82	49.05 ± 4.63^**^	34.78 ± 2.22^b^
	geosmin	20.00 ± 4.80	63.57 ± 5.17^***^	54.52 ± 1.48^c^
	hexanal	29.13 ± 5.96	59.81 ± 4.89^**^	37.19 ± 5.19^b^
	delta-octalactone	62.63 ± 4.29	20.60 ± 3.08^***^	–63.93 ± 5.93^a^

### 3.2. Laboratory responses of *Glossina pallidipes* to CO_2_

Proportion of *G. pallidipes* tsetse flies and the distances they flew within the CO_2_ treated arm of the olfactometer was significantly (*p* < 0.05) higher than those in the control arm (purified air) when 3.75%, 5.00%, 6.25%, 7.50% or 8.75% of the CO_2_ was dispensed (figure 1). We observed dose-dependent increased responses (attraction) of *G. pallidipes* to CO_2_. We observed the greatest responses (attraction) of *G. pallidipes* (25.90% ± 3.21%) at 6.25% CO_2_ (figure 1). Our independent *t*-test showed that the distances flown by *G. pallidipes* on the treated arm (*M* = 60.02, s.d. = 2.86 cm) were greater than similar responses in the control arm (*M* = 37.03, s.d. = 1.41 cm) on dispensing 6.25% CO_2_, *t*_4_ = 14.54, *p* = 0.0001 (figure 1). Higher dosages of 10.00%, 11.25% or 12.50% CO_2_ significantly (*p* < 0.05) increased proportions of flies in the treatment arm and distances flown in the arm with treated than without (figure 1). We observed peak repellence of *G. pallidipes* (–21.44% ± 3.58%) on exposure to CO_2_ at a concentration greater than 11.25% (figure 1). Our independent *t*-test revealed that distances flown by *G. pallidipes* on the control arm (*M* = 52.37, s.d. = 3.52 cm) were significantly higher than similar distances flown in the treated arm (*M* = 25.73, s.d. = 2.69 cm) on dispensing 11.25% CO_2_, *t*_4_ = 11.82, *p* = 0.0003 (figure 1).

**Figure 1.**
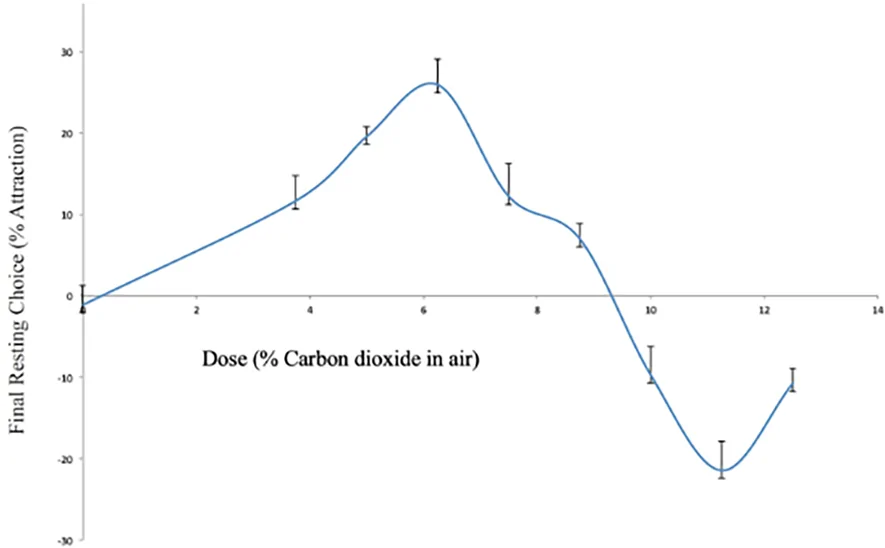
Mean laboratory responses of *G. pallidipes* to CO_2_ at varying doses. Bars show mean ± s.e.m., *n* = 3 treatments per treatment. Individual raw values are provided in the supplementary material.

### 3.3. Field responses of *Glossina pallidipes* to geosmin and CO_2_

We confirmed responses (attraction) of *G. pallidipes* to geosmin and CO_2_ in the field using Latin square experimental design. We collected 1724 *G. pallidipes*, comprising 552 males (82 tenerals and 470 non-tenerals) and 1172 females (138 tenerals and 1034 non-tenerals) during the field experiment. We confirmed normal distribution of *G. pallidipes* catches for both males (*W* = 0.95, *p* = 0.41) and females (*W* = 0.95, *p* = 0.45), and variances did not differ significantly (*F*_15,15_ = 0.90, *p* > 0.05) using the Shapiro–Wilk tests, and separately analysed male and female catches owing to significant (*t*_30_ = −2.10, *p* = 0.004) differences in responses of males and females to the treatments.

In the Latin square experiment for males, no significant day × site, day × treatment or site × treatment interactions were detected (*p* > 0.05), allowing pooling across sites. NG2G traps baited with geosmin, CO_2_ (5 l min^–1^) and POCA captured significantly more males than unbaited controls (*p* < 0.05; figure 2), increasing catches by 4.2-, 6.6-, and 9.8-fold, respectively (figure 2). Catches in CO_2_-baited traps did not differ significantly from POCA or geosmin (figure 2), although POCA outperformed geosmin (*p* < 0.05; figure 2).

**Figure 2.**
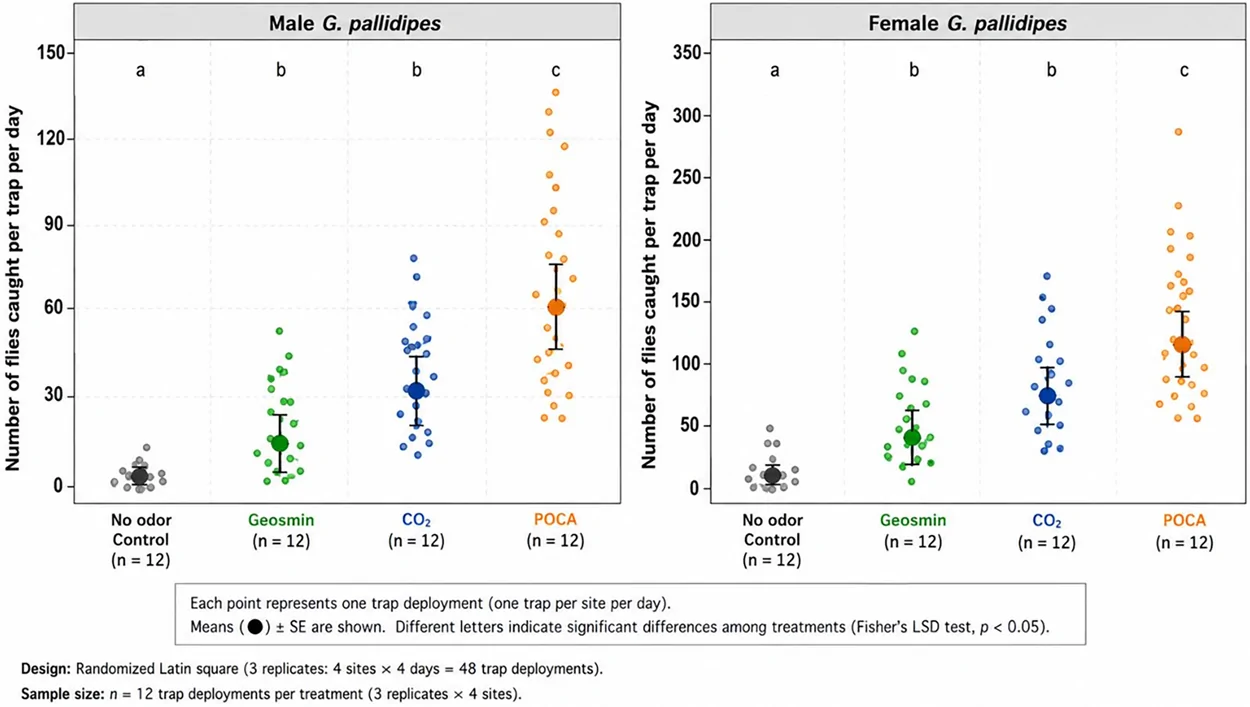
Mean *G. pallidipes* catches on NG2G baited with geosmin and CO_2_. Raw values are provided in the supplementary material.

Equally for females, no significant day × site, day × treatment or site × treatment interactions were detected (*p* = 0.26, 0.15 and 0.15, respectively), and data were pooled across sites. NG2G traps baited with geosmin (44.43; 95% confidence interval (CI), 16.88–114.45), CO_2_ (76.64; 95% CI, 33.28–174.89) and POCA (127.24; 95% CI, 69.47–232.39) captured significantly more females than un-baited controls (13.23; 95% CI, 4.31–37.15; *p* < 0.05; figure 2), representing 3.4-, 5.8- and 9.6-fold increases, respectively (figure 2). Across all treatments, female catches were significantly higher than male catches (*p* < 0.05; figure 2). The mean field release rate of geosmin was 1.07 ± 0.02 mg h^–1^, whereas CO_2_ was maintained at 6.25 l min^–1^.

## 4. Discussion

Understanding and manipulating tsetse behaviour is central to control of African trypanosomosis in humans and livestock, and to reducing the nuisance of biting. Odour-baited traps and targets form the backbone of vector management by attracting and killing flies, hence suppressing populations and diverting them from hosts to facilitate interruption of transmission. When integrated into control programmes, bait technologies provide cost-effective population reduction, and their combination with repellents in a ‘push–pull’ strategy offers rapid, area-wide suppression with enhanced operational efficiency [13]. Advancing this approach depends on identification and characterization of potent chemical cues that reliably mediate attraction or avoidance, thereby expanding and optimizing the tsetse control toolbox. The currently used tsetse attractant (POCA) is about 20% attractive as compared with the natural cattle host, thus permitting tsetse flies to still feed on cattle hosts instead of being lured to traps and targets. There is a need to improve the efficiency of POCA or come up with new candidates that are highly effective.

In the present study, laboratory assays show that *G. pallidipes* exhibits dose-dependent attraction to geosmin, 2-pentanol or hexanal, with geosmin eliciting the greatest response. Flies exposed to geosmin consistently flew longer distances upwind and preferred the treated arm relative to controls, indicating that geosmin is a potent olfactory cue. Although 2-pentanol and hexanal also attracted flies, the responses were weaker, highlighting differences in tsetse sensitivity to structurally distinct volatiles. Interestingly, the behavioural response of *G. pallidipes* to geosmin is opposite to that observed in *D. melanogaster*, where geosmin acts as a strong repellent associated with microbial contamination [15]. This divergence underscores the species-specific nature of olfactory perception and the need to test odorants directly in tsetse flies rather than relying on model dipterans. Similar to the tsetse case found in this study, other insects, such as *A. aegypti* mosquitoes, are attracted to geosmin, which the mosquito uses to select microbe-rich sites for oviposition that support larval development. The apparent interspecific variation suggests that the ecological significance of geosmin is context-dependent, and that *G. pallidipes* may interpret geosmin as a positive cue, potentially indicating environments favourable for survival or feeding. This further suggests that tsetse flies may be attracted to sites such as dumps or fermenting fruit, where geosmin is produced by microbial activity. Although the field performance of geosmin was less than that of POCA, its value lies in expanding the repertoire of behaviourally active compounds available for tsetse control. Because geosmin represents a microbial-derived cue rather than a traditional host-derived odour, it may contribute complementary information when combined with existing attractants such as POCA and CO_2_. Such combinations may improve trap performance, species specificity or lure longevity. These possibilities warrant future optimization studies. The practical value of geosmin lies in its ability to enhance POCA rather than compete with it. Although POCA produced higher field catches, geosmin represents a chemically distinct microbial-derived cue that broadens the range of known behaviourally active compounds for *G. pallidipes*. Its greatest potential lies in improving multi-component blends, identifying conserved olfactory pathways, and informing the development of species-specific attractants. We acknowledge that commercially available geosmin is currently more expensive than conventional POCA components, and its operational deployment would require formulation strategies that improve stability, controlled release rate for reduced costs, and demonstrate synergistic effect benefits in field performance.

Interestingly, the functional organization of the tsetse olfactory system is very much similar to that of *D. melanogaster*—a fly whose olfactory system has been best characterized. The sensillum types in tsetse especially the *Glossina morsitans morsitans* species and *D. melanogaster* are morphologically similar, suggesting functional conservation [27]. However, sensillum types in different insects respond to different chemical cues. For instance, in *Drosophila*, the trichoid sensilla respond to pheromones [28], whereas in *G. m. morsitans*, these sensilla respond to a wide variety of compounds [29]. Furthermore, owing to the differing food preferences between fruit fly and tsetse fly, specific odorant receptors and their expressions can differ, reflecting their distinct ecological roles. These remarkable converse observations in behavioural responses of tsetse to *Drosophila* cues may be owing to differences in their olfactory perception, suggesting that we can potentially get compounds that will provide enhanced protection of humans and their livestock from tsetse and trypanosomosis from sources emitting attractive compounds. This further indicates that chemical compounds eliciting a response in *Drosophila* will also give a response to tsetse flies, which may be similar or different.

Similar to geosmin, CO_2_ showed a dose-dependent effect on tsetse behaviour. Moderate concentrations (3.75%–6.25%) elicited the strongest attraction, with flies flying longer upwind and preferring the treated arm, peaking at 6.25% CO_2_. This confirms CO_2_ as a biologically relevant cue, by contrast to *Drosophila*, where it triggers avoidance [30]. Higher concentrations (more than 10%) became repellent, with flies favouring the control arm, producing an inverted-U response, a phenomenon previously observed by Torr [31]. There was no *G. pallidipes* response to approximately 9% CO_2₂_ that was similar to purified air (78% nitrogen, 21% oxygen and 0.03% CO_2_) as indicated by the *x*-intercepts in figure 1. Such patterns reflect a general principle in insect olfaction: odorants can switch from attractive to aversive at high doses owing to recruitment of additional olfactory neurons or receptor saturation. In mosquitoes, for example, high concentrations of attractive odorants activate more antennal neurons, including those signalling repellency [32]. Similarly, in *Drosophila* and *Anopheles*, high odorant levels can trigger extra receptors, causing central circuits to interpret the signal as aversive and shift behaviour from attraction to avoidance [32].

Across all treatments, female *G. pallidipes* were caught in significantly higher numbers than males. This stronger female attraction to geosmin, CO_2_ and POCA probably reflects sex-specific differences in olfactory sensitivity and behaviour. Because females need blood meals for reproduction, they generally show stronger host-seeking tendencies and may respond more intensely to host- or environment-related odour cues [33,34]. Sex differences in olfactory receptor and odorant-binding protein expression, reported in other dipterans such as *Anopheles* [32], may also contribute to this heightened sensitivity. In addition, sex-specific chemosensory circuits and semiochemical signalling, including pheromone pathways, can shape male and female responses differently [35]. These differences have practical implications: if females respond more strongly to attractants, traps can be optimized to target them, which is valuable because females drive population growth and disease transmission. Still, a better understanding of sex-specific olfactory receptors, potentially through transcriptomics or functional assays, is needed to design blends that work effectively for both sexes.

Finally, this study shows that *G. pallidipes* exhibits distinct and quantifiable behavioural responses to a range of volatile compounds, including molecules reported as repellents in other dipterans such as *Drosophila*. The observed upwind flight orientation, resting preferences and dose-dependent effects highlight the importance of empirical testing in tsetse rather than extrapolating from model organisms. These results broaden the chemical landscape beyond traditional host-derived odour blends and provide a practical basis for identifying additional cues capable of modulating tsetse behaviour. This approach may also help address the limited availability of effective attractants and repellents for riverine (palpalis group) species such as *Glossina fuscipes fuscipes*.

## 5. Conclusion and future directions

Our results show that geosmin strongly attracts *G. pallidipes* in a dose-dependent way, suggesting that tsetse flies are highly sensitive to this chemical. Although the exact receptors are not yet known, future molecular and electrophysiological studies can identify the pathways involved and help design better attractants or repellents. Additionally, field-testing mixtures of geosmin, CO_2_ and POCA will show whether these combinations improve trap performance and whether they work across other tsetse species, including the *palpalis* group for which potent and validated attractants are currently lacking. Similar testing in species such as *Glossina austeni*, *Glossina morsitans centralis* and *Glossina swynnertoni* will help refine integrated control strategies. Future field studies should evaluate hexanal and 2-pentanol individually and in combination with existing attractant blends to determine whether their moderate laboratory attraction translates into improved field performance.

For practical field use, improving chemical release systems is essential. Current sachet dispensers for synthetic phenols in the POCA blend [36] could be optimized, and micro-encapsulation methods, such as *β*-cyclodextrin, may provide longer-lasting, controlled release. Repellents in a ‘push–pull’ strategy should ideally be dispensed from hosts at rates sufficient for extended activity; for example, a repellent blend has been successfully micro-encapsulated for thermal stability and slow release [25,37]. However, micro-encapsulation of attractants and their field performance remain untested. Evaluating tsetse responses to a chemical fingerprint of geosmin, CO_2_ and POCA could clarify the incremental effects of each component. Optimizing delivery systems for attractant and repellent blends may enhance their practical application in vector control.

## Supplementary Material



## Data Availability

Summarized data are included in the article, and all raw data underlying table 1, and figures 1 and 2 are available in the supplementary material [38].
